# Augmented reality-aided unicompartmental knee arthroplasty

**DOI:** 10.1186/s40634-022-00525-4

**Published:** 2022-09-05

**Authors:** Sachiyuki Tsukada, Hiroyuki Ogawa, Kenji Kurosaka, Masayoshi Saito, Masahiro Nishino, Naoyuki Hirasawa

**Affiliations:** Department of Orthopaedic Surgery, Hokusuikai Kinen Hospital, 3-2-1 Higashihara, Mito, Ibaraki 310-0035 Japan

**Keywords:** Knee, Computer-assisted surgery, Augmented reality, Virtual reality, Smartphone

## Abstract

**Purpose:**

To illustrate a surgical technique for augmented reality (AR)-assisted unicompartmental knee arthroplasty (UKA) and report preliminary data.

**Methods:**

We developed an AR-based navigation system that enables the surgeon to see the tibial mechanical axis superimposed on the patient’s leg in addition to the tibial cutting angle. We measured the tibial resection angle in 11 UKAs using postoperative radiographs and calculated the absolute difference between preoperative target angle and postoperative measured angle. The target angle was determined for each patient: mean values were 0.7° ± 1.0° varus in coronal alignment and 5.3° ± 1.4° posterior slope in sagittal alignment.

**Results:**

The angles measured on postoperative radiographs were 2.6° ± 1.2° varus in the coronal plane and 4.8° ± 2.5° posterior slope in the sagittal plane. The absolute differences between the target and measured angles were 1.9° ± 1.5° in coronal alignment and 2.6° ± 1.2° in sagittal alignment. No patients experienced complications, including surgical site infection and periprosthetic fracture.

**Conclusion:**

The AR-based portable navigation system may provide passable accuracy in terms of proximal tibial resection during UKA.

**Level of Evidence:**

IV

## Introduction

Unicompartmental knee arthroplasty (UKA) is an attractive option to treat unicompartmental end-stage knee osteoarthritis and osteonecrosis [15]. Accurate tibial bone cutting is one of the most essential determinants for obtaining proper limb alignment after UKA [9]. Malalignment of the lower limb can increase the risk of postoperative complications including contralateral compartmental osteoarthritis, component loosening, and component wear, following UKA [13].

Computer-assisted navigation systems have contributed to improvement of the accuracy of bone cutting in knee arthroplasty. These navigation systems can be divided into three groups: *image-based large-console navigation*, *imageless large-console navigation*, and *portable navigation* [7]. Both imageless large-console navigation and portable navigation avoid radiation exposure as they do not require preoperative imaging, such as computed tomography [7]. The surgeon can employ a portable navigation system without adding significant extra cost because it does not require expensive equipment [10].

Augmented reality (AR) technology shows great promise in computer-assisted surgery due to its unique ability to fuse live images and synthetic computer-generated images [4, 8]. We developed a portable navigation system applied to AR technology that projects the tibial mechanical axis and the varus and posterior slope angles of tibial cutting guide on real world through a smartphone display [18]. This is the first study of tibial bone cutting during UKA assisted with AR technology. The aim of this preliminary clinical study was to assess the accuracy of the AR-based portable navigation system for proximal tibial resection during UKA.

## Methods

This retrospective single-arm cohort study was approved by the institutional ethics board. We reviewed 11 consecutive UKAs performed using the AR-based portable navigation system to proximal tibial resection between January 2020 and November 2021. All patients provided written informed consent.

All surgeries were performed by one surgeon (ST) using a Persona Partial Knee System (Zimmer-Biomet, Warsaw, IN). Tibial bone resection was performed using the AR-based portable navigation system. The target angle of tibial resection was determined for each patient using the original varus angle and posterior slope of the proximal tibia as references. In the coronal plane, we aimed to resect the proximal tibia perpendicular to the mechanical axis of the femur except in patients with preoperative varus angle of the proximal tibia ≥ 6°, in whom we set the target varus angle to 2° or 3° because varus alignment exceeding 4° was reported to be associated with translation in the mediolateral direction [16]. In the sagittal plane, we aimed to resect the proximal tibia with the same angle of the preoperative posterior slope of the medial compartment in each patient. However, we reduced the target angle in patients with a preoperative posterior slope ≥ 6° because excessive posterior slope has been shown to be associated with greater tension of the anterior cruciate ligament and excessive translation in the mediolateral direction [16]. Following tibial bone resection, the distal femur was resected according to the spacer block technique. All prostheses were implanted using Simplex P bone cement (Stryker, Mahwah, NJ).

The tibial resection angle was measured using a standing long-leg radiograph with ImageJ software (US National Institutes of Health, Bethesda, MD). The coronal alignment was measured with reference to the perpendicular line connecting the midpoint of the tibial plateau and the midpoint of the tibial plafond [17]. The sagittal alignment was measured with reference to the perpendicular line connecting the anterior one third of the medial tibial plateau and midpoint of the tibial plafond [17]. Angles were recorded to two decimal places and rounded off to one decimal place. The accuracy of the AR-based navigation system was assessed by calculating the difference between the preoperative target angle and postoperative measured angle. Any complications were recorded with special attention to periprosthetic fracture.

### Surgical technique of tibial bone resection using AR-based portable navigation system

AR technology projects digital information onto the real world [11, 12]. The AR-based navigation system enables the surgeon to see the tibial mechanical axis on the patient’s leg and the tibial cutting angle in real-time.

**Fig. 1:**
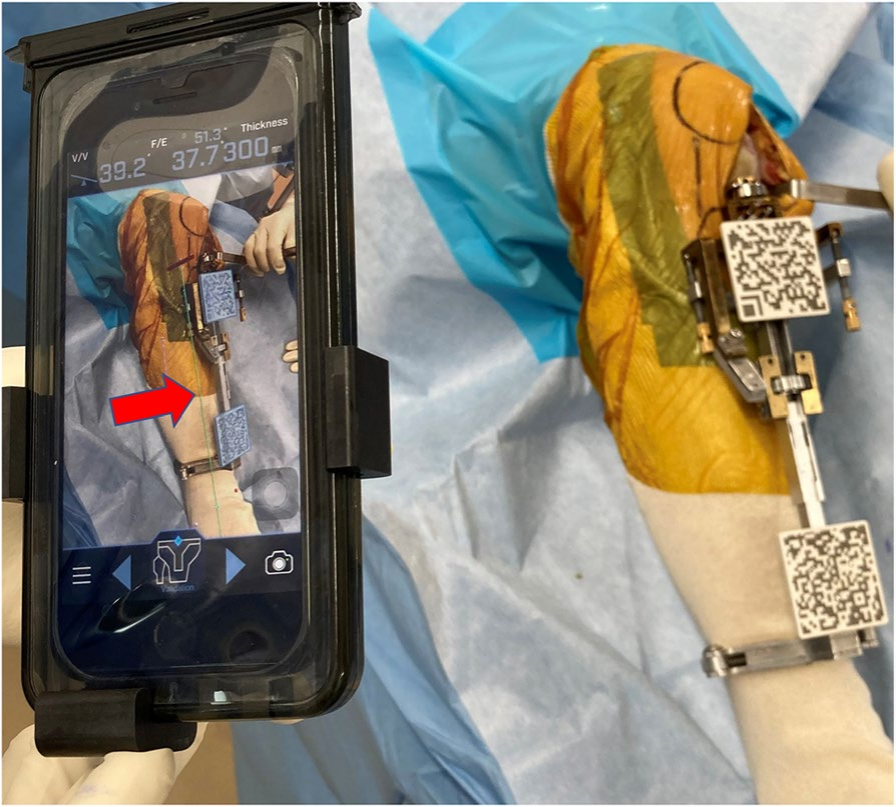
AR-based portable navigation system enables the surgeon to see the tibial mechanical axis in the surgical field through the smartphone (green line indicated by red arrow). On the smartphone display, the color of the marker turns blue after the smartphone camera recognized the QR code. The extramedullary tibial cutting guide carries two markers with QR codes

**Fig. 2:**
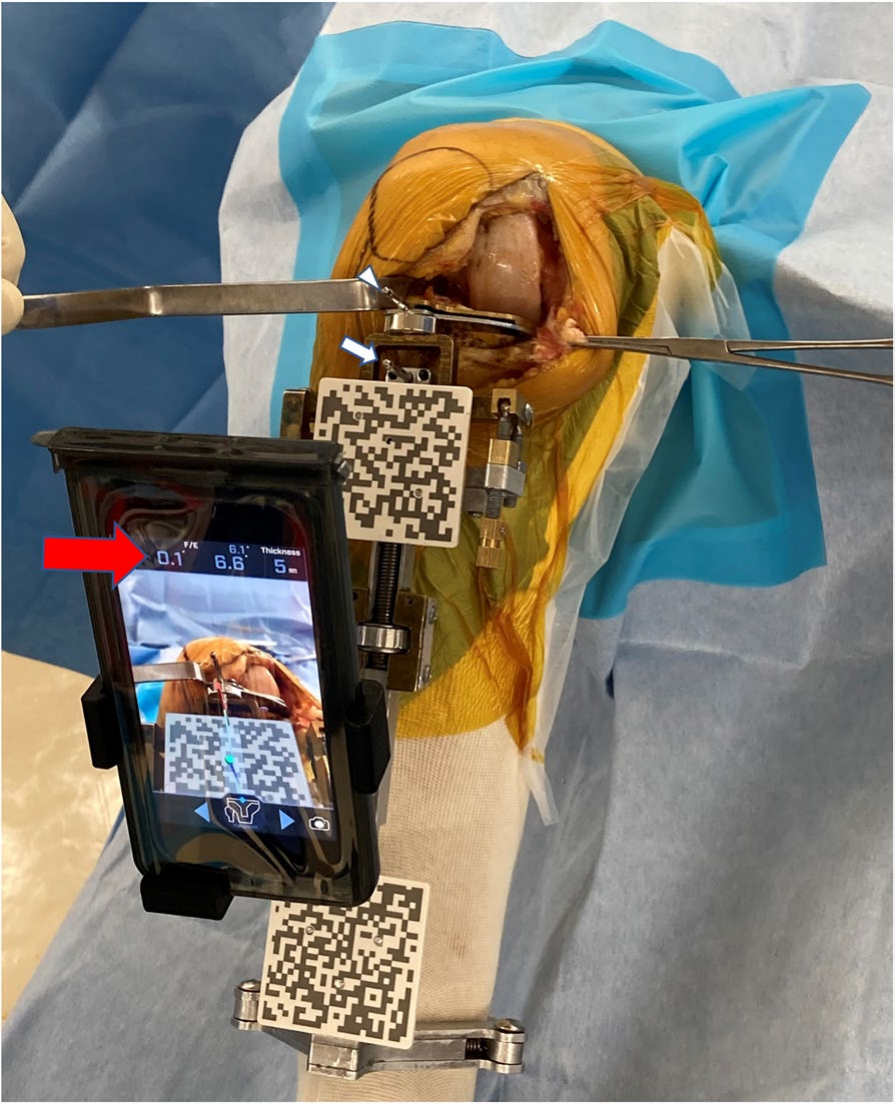
The surgeon aligns the cutting block of the proximal tibia while viewing the varus/valgus alignment, posterior slope, and medial resection depth on the smartphone display. Based on preoperative planning, the surgeon set the cutting block on the varus angle of 0.1°, posterior slope of 6.6°, and medial depth of 5 mm (red arrow). First, one fixation pin (white arrow) is inserted parallel to the anteroposterior axis to fix the extramedullary guide. Second, another pin (white arrowhead) is inserted to fix the cutting block. Note that no extra pins are required to attach the sensor of the AR-based navigation system compared with standard extramedullary guide and cutting block

The extramedullary tibial cutting guide of the AR-based navigation system carries two markers with Quick Response (QR) codes (Figs. 1 and 2). The extramedullary tibial cutting guide of the AR-based navigation system was set on the patient’s lower leg in a similar manner to the standard cutting guide. First, the ankle clamp was placed proximal to the malleolar. Second, the surgeon inserted one pin to fix the cutting guide parallel to the anteroposterior axis of the tibia because the AR-based navigation system was programmed to recognize the direction of the pin as the anteroposterior axis (Fig. 2).

**Fig. 3:**
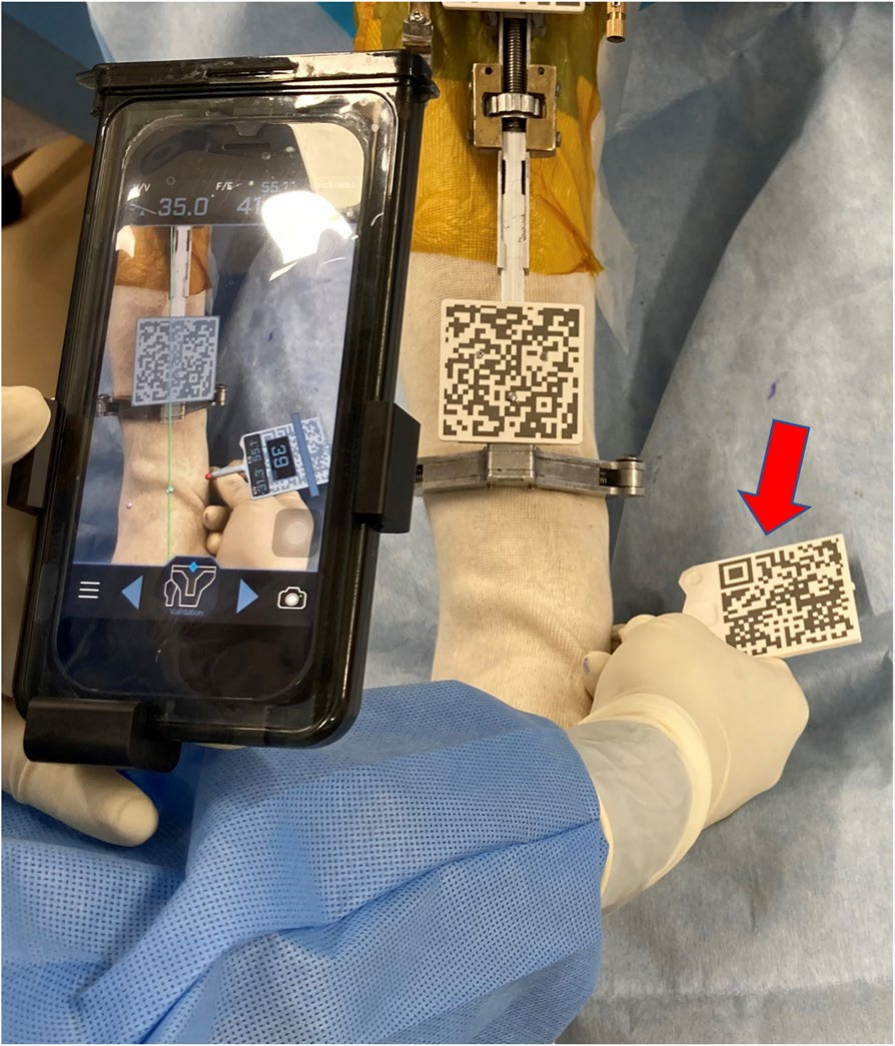
Registration of the medial malleolus using a pointer marked with a QR code (red arrow). Note that the AR-based portable navigation system works if the smartphone recognizes only one of the two QR codes

**Fig. 4:**
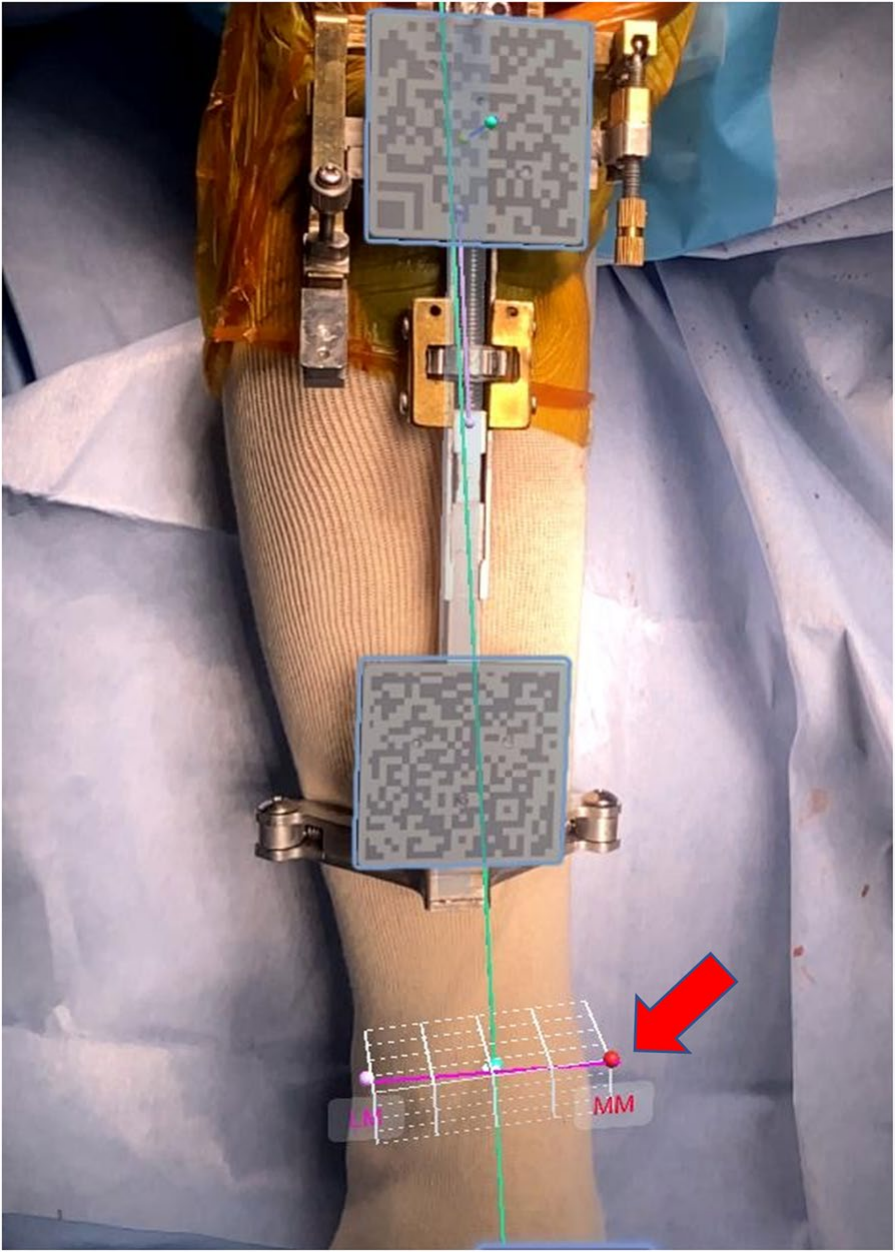
Awareness of inappropriate registration. As the AR-based navigation system can visualize the registration point, the surgeon can easily recognize which point is inappropriate. The registration point of medial malleolus floats on the patient’s leg in this patient (red arrow)

The sensor of the AR-based navigation system is the camera of the smartphone. The smartphone camera recognizes the QR code of the extramedullary tibial cutting guide. Using a pointer marked with a QR code, the surgeon registers three bony landmarks: the most prominent point of the medial malleolus, the most prominent point of the lateral malleolus, and the tibial center on the tibial plateau (Fig. 3). Visualization of registration points is a major advantage of the AR-based navigation system, which enables surgeon to easily recognize whether the registered point is wrong or not (Fig. 4). The navigation system creates a 3-dimensional tibial coordinate system to express the position of the tibial cutting guide. The three lines constituting the 3-dimensional coordinate system of AR-based navigation are: (1) the tibial mechanical axis; (2) the tibial anteroposterior axis; and (3) the cross product of these two tibial axes. The tibial mechanical axis is defined as the line connecting the center of the ankle and the tibial center on the tibial plateau. The registration of medial compartments of the tibial plateau is used for the resection level of the tibia, allowing the surgeon to view the proximal tibia resection level.

After completing registration, the AR-based navigation system enables the surgeon to view the reference lines superimposed on the tibia on their smartphone display (Fig. 1). The surgeon can also view the angles of varus/valgus and posterior slope on the display (Fig. 2). The surgeon fixes the tibial resection block while viewing these angles. As the guide pinhole below the tibial prosthesis can be a stress point increasing the risk of tibial stress fracture [1], the cutting block of the AR-based navigation system has only one pinhole that is located at the cross point of the vertical and horizontal bone cut lines (Fig. 2). Following fixation of the tibial resection block, the surgeon cuts the proximal tibia in the standard manner.

**Fig. 5:**
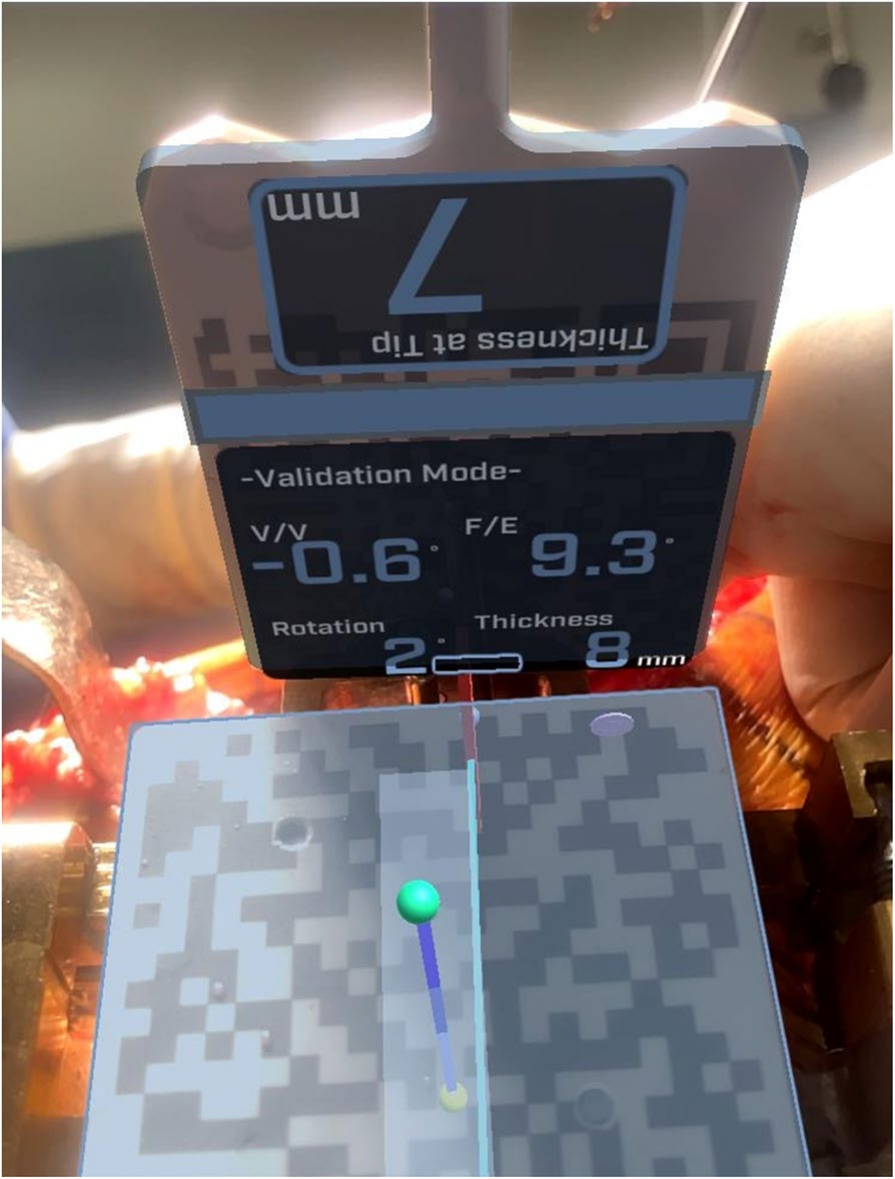
Verification of tibial bone cutting. The AR-based navigation system allows the surgeon to confirm the actual cutting angle and depth of resection

After tibial bone resection, the AR-based navigation system allows the surgeon to verify the actual cutting angle and depth of resection. The display of the smartphone shows varus/valgus angle, tibial slope angle, rotation angle, and the thickness of bone resection (Fig. 5).

## Results

The patient characteristics and results of radiographic measurement of the patients are summarized in Table 1.

**Table 1:** Patient characteristics and results of radiographic measurement

Patient	Side	Sex	Age, year	Height, cm	Weight, kg	BMI, kg/m^2^	Diagnosis	Preoperative tibial varus angle, °	Target varus angle, °	Postoperative varus angle, °	Preoperative posterior slope angle, °	Target posterior slope, °	Postoperative posterior slope angle, °
1	Right	Male	79	168	70.4	25.0	OA	8	2	3.4	4	4	5.3
2	Left	Male	79	168	70.4	25.0	OA	6	2	2.0	2	2	3.3
3	Left	Male	72	163	68.9	25.9	OA	4	0	3.3	5	5	1.6
4	Right	Male	77	156	55.9	22.9	AVN	5	0	3.9	10	7	1.8
5	Right	Female	82	149	53.0	24.0	AVN	3	0	1.3	7	6	8.8
6	Right	Female	81	150	57.0	25.3	OA	4	0	1.4	13	7	5.0
7	Right	Female	86	157	52.8	21.5	OA	3	0	2.4	8	6	2.6
8	Right	Male	87	159	67.0	26.7	OA	4	0	4.7	7	6	3.3
9	Right	Female	83	142	52.0	25.8	OA	5	0	0.8	5	5	6.8
10	Left	Female	83	142	52.0	25.8	OA	7	2	3.3	7	5	6.7
11	Left	Female	84	151	50.4	22.1	OA	6	2	2.4	6	5	8.0

*AVN* Avascular necrosis, *BMI* Body mass index, *OA* Osteoarthritis

With regard to coronal alignment, the mean ± standard deviation of postoperative varus angle was 2.6° ± 1.2°. With regard to sagittal alignment, postoperative posterior slope angle was 4.8° ± 2.5°.

The absolute differences between the preoperative target resection angles and postoperative measured angles were 1.9° ± 1.5° in coronal alignment and 2.6° ± 1.2° in sagittal alignment.

No patients experienced complications, including surgical site infection and periprosthetic fracture.

## Discussion

We reported the surgical technique of AR-based portable navigation for tibial bone resection during UKA. AR technology has attracted increasing interest in surgical practice [2]. In orthopedic surgery, AR has been applied to a wide spectrum of procedures, such as total knee arthroplasty (TKA), total hip arthroplasty, tumor resection, arthroscopic surgery, and fracture treatment [2, 5, 6, 8, 11, 12, 14, 18–20]. This is the first report applying AR technology to UKA (Table 2).

**Table 2:** Studies on the use of AR technology in knee arthroplasty

Reference	Procedure	Characteristics of the AR technology-assisted system	Required preoperative imaging study	Radiographic outcomes
Pokhrel, et al. [14]	TKA	Superimposing the image of the remaining area of bone to resect onto the actually resected bone surface in the surgical field	Computed tomography scan of the knee	No radiographic data available
Tsukada, et al. [18]	TKA	Superimposing the tibial mechanical axis and rotational axis onto the patient's limb in the surgical field and showing the resecting angles and depth in real time	Nothing	Cutting error of proximal tibia was less than 1° in both coronal and sagittal planes and less than 2° in rotational alignment
Iacono, et al. [6]	TKA	Superimposing the tibial and femoral mechanical axes onto the patient's limb in the surgical field and showing the resecting angles in real time	Nothing	Cutting error of both proximal tibia and distal femur was less than 1° in coronal plane and less than 2° in sagittal plane, respectively
Tsukada, et al. [19]	TKA	Superimposing the femoral mechanical axis and location of the femoral head center onto the patient's limb in the surgical field and showing the resecting angles in real time	Nothing	Cutting error of distal femur using AR technology was significantly smaller than that using conventional intramedullary rod technique
Fucentese, et al. [5]	TKA	Showing the bone resecting angles and the lengths of the medial collateral ligament and lateral collateral ligament in real time	Computed tomography scans of the hip, knee and ankle	No radiographic data available
Current study	UKA	Superimposing the tibial mechanical axis and rotational axis onto the patient's limb in the surgical field and showing the resecting angles and depth in real time	Nothing	Cutting error of proximal tibia was less than 2° in coronal plane and less than 3° in sagittal plane, respectively

*AR* Augmented reality, *TKA* Total knee arthroplasty, *UKA* Unicompartmental knee arthroplasty

There are several advantages to using AR-based portable navigation for UKA. First, the AR-based navigation system can visualize the mechanical axis and registration points. Other navigation systems do not have this capability. The visualization may be useful not only for enhancing accuracy of surgical procedures, but also in surgical education for inexperienced surgeons.

Second, the running costs for use of the AR-based portable navigation in UKA are low. The AR-based navigation system can be used with an application downloaded to the surgeon’s own smartphone, several QR code markers, and the dedicated extramedullary guide. In addition, neither an assistant nor additional equipment is required in the nonsterile zone because the operation of the AR-based navigation system is accomplished by the surgeon in the sterile zone alone.

Third, no extra pins are needed to use the AR-based navigation system in UKA because the extramedullary cutting guide carries QR codes for registration. Pinholes located in the proximal tibial plateau are associated with risk of periprosthetic fracture [3]. Therefore, avoiding the insertion of extra pins to attach the navigation sensor may reduce the risk of periprosthetic fracture.

This study had several limitations. First, this was a preliminary study in a single-arm cohort with small sample size. Although a pilot study was crucial to assess the feasibility of a larger comparative study, it must be noted that this study could not conclusively demonstrate the clinical effectiveness of the AR-based navigation system. Second, the results lack generalizability because all surgeries were performed by a single surgeon at a single institute, and surgeon’s experience has been shown to affect the clinical and radiographic outcome of UKA [21].

## Conclusions

The AR-based navigation system may be an effective option to enhance the accuracy of tibial bone resection during UKA. This preliminary study provided important information for future studies to investigate the clinical effectiveness and safety of the AR-based navigation system.

## Data Availability

The datasets used and/or analysed during the present study are not publicly available. Data are, however, available from the corresponding author on reasonable request.

## Abbreviations

AR: Augmented Reality
QR: Quick Response
TKA: Total Knee Arthroplasty
UKA: Unicompartmental Knee Arthroplasty
